# Lobe-wise cognitive load detection using empirical Fourier decomposition and optimized machine learning

**DOI:** 10.3389/fphys.2025.1700756

**Published:** 2026-01-08

**Authors:** Kunamneni Chervitha, Lakhan Dev Sharma

**Affiliations:** School of Electronics Engineering, VIT-AP University, Guntur, Andhra Pradesh, India

**Keywords:** cognitive load, lobe-wise, electroencephalogram, empirical Fourier decomposition, optimized machine learning

## Abstract

**Introduction:**

Cognitive load significantly affects neural activity, making its assessment important in neuroscience and human–computer interaction. EEG provides a noninvasive way to monitor brain responses to mental effort. This study explores EEG-based feature extraction and classification methods to accurately assess cognitive load during mental tasks.

**Methods:**

EEG signals were recorded from all brain lobes over 4 seconds and decomposed into ten intrinsic mode functions using Empirical Fourier Decomposition (EMFD). Entropy-based features were extracted, and feature reduction was applied. Both lobe-wise and overall classifications were performed using optimized ensemble machine learning (OML) and conventional ML classifiers. The approach was evaluated on the Mental Arithmetic Task (MAT) and Spatial Transcriptomic Multi-View (STEW) datasets.

**Results:**

The proposed EMFD-based OML framework achieved high accuracy, reaching 97.8% on the MAT dataset and 96.4% on the STEW dataset. Lobe-wise analysis showed strong performance across all brain regions, with the frontal lobe achieving the highest accuracies of 97.8% (MAT) and 96.08% (STEW).

**Discussion:**

The findings demonstrate that EMFD combined with optimized ensemble learning effectively enhances EEG-based cognitive load detection. The consistent performance across datasets confirms the robustness of the method, while lobe-wise analysis highlights the frontal lobe’s key role in cognitive processing. The proposed framework outperforms existing methods and shows strong potential for real-world cognitive monitoring applications.

## 1 Introduction

Electroencephalography (EEG) directly measures brain activity through the recording of electrical signals produced by neurons. EEG is a reliable tool for assessing workload due to its high temporal resolution in measuring brain responses to cognitive demands (Teplan et al., 2002). EEG provides a direct measurement of neural correlates linked to mental effort, including variations in frequency bands. Prior studies indicate that distinct EEG patterns are associated with varying levels of workload, establishing EEG as an effective instrument for real-time cognitive assessment (Antonenko et al., 2010; Yedukondalu et al., 2025b).

Tasks require a range of cognitive resources, including specialized attention, knowledge, decision-making abilities, and working memory. Human brains possess a finite ability for processing and integrating data. Cognitive load (CL) refers to the workload placed on limited cognitive resources (Antonenko et al., 2010). In neuroscience, the continuous assessment of CL is often demonstrated to affect neural transitions. Scholars frequently investigate a range of physiological parameters, including pupil dilation (Deschênes et al., 2015), ocular metrics, facial expressions, and heart rate (Duman, 2014), to assess cognitive load. Stress can disrupt balance among parasympathetic and sympathetic neural systems, leading to increased reactivity of the parasympathetic network (Wielgosz et al., 2016). This deregulation may lead to adaptive changes, including alterations in blood pressure, respiration, skin conductance, and heart rate variability (He et al., 2019; Oliveira Filho et al., 2023; Abdulwahhab et al., 2024). Assessing neural activity during tasks with EEG signals helps assess CL in real time. This technology aids in assessing the cognitive load associated with neurological conditions like dementia and brain trauma in healthcare (Tasci et al., 2023; Yedukondalu et al., 2025a).

EEG is a valuable tool for exploring and quantifying various psychological workloads and functions, greatly improving cognitive neuroscience and psychology. This method is essential for monitoring and evaluating cognitive processes through noninvasive assessment of functional brain changes. The extraction of sought details via EEG data preprocessing entails feature extraction, feature selection, and classification. Various methods for classifying EEG-based information have been reported in the literature, demonstrating a significant influence on the medical field (Dedovic et al., 2005; Wan et al., 2023; Boaretto et al., 2023).

Wang and Sourina (2013) developed a method to evaluate CL by focusing on neurological responses over mental arithmetic assignments, utilizing the fractal dimension spectrum. Zarjam et al. (2012) presented an approach for determining mental load in relation to various levels of mental arithmetic demands. Despite these advancements, the accuracy of noninvasive stress assessment methods remains insufficient and requires enhancement. Shon et al. (2018) used a strategy employing genetic algorithms (GAMs) to select stressful attributes, optimize model parameters, and enhance stress identification. Sharma et al. (2021a) developed a Bayesian-optimized K-nearest neighbors (KNN) classifier utilizing EEG signals to detect cognitive stress via mental arithmetic tasks. The study achieved sample entropy characteristics with an accuracy of 96% through the stationary wavelet transform (SWT) and a Savitzky–Golay filter. Using wavelet packet transform (WPT) and random forest, Zhang and Min (2020) successfully categorized four human emotions based on differential entropy (DE) features with an accuracy of 87.3%. Sadiq et al. (2021) put forth an automated framework that employs multi-domain attributes and feature selection to enhance motor-imagery EEG categorization. Yu et al. (2021) introduced a prototype that employs enhanced neural networks and improved Fourier decomposition (IEFD) to classify mental imagery and EEG motor tasks, demonstrating high accuracy. Komolovaitė et al. (2022) developed convolutional neural network (CNN) models, specifically EEGNet, for categorizing emotion types and facial inversion stimuli. A generalized mixture model (GMM) for identifying emotions was proposed by Krishna et al. (2019), utilizing EEG signals to assess emotional conditions of individuals with mental disabilities.

Krishna et al. (2019) utilized EEG to assess the emotional states of individuals with mental disabilities and presented a GMM for emotion recognition. Safari et al. (2024) proposed a strategy for assessing CL by analyzing neurodynamic causality using spatial transcriptomic multi-view (STEW)-EEG data. Brain connectivities were obtained and refined using a hierarchical feature-picking process. Deep neural networks were applied to estimate mental workload via EEG. The study utilized the direct directed transfer function (dTDTF) technique to analyze brain connectivity and employed a deep hybrid model that integrates CNN and LSTM. This approach resulted in an accuracy of 83.12% for classifying workload levels via patient-free methodology on the STEW dataset (Safari et al., 2024). Aksu et al. (2024) integrated EEG and eye-tracking information to categorize mental workload over n-back tasks through machine learning techniques. The study involved 15 participants and achieved an accuracy of 76.59% in classifying four workload categories, utilizing 34 selected attributes. It underscores the synergy between EEG and visual tracking for estimating workload. Akbari et al. (2021) used geometrical attributes for early identification of depression and achieved an accuracy of 98.79% with KNN, exceeding that of traditional methods. Sadiq et al. (2020) demonstrated that motor-imagery task categorization in BCI could reach an accuracy of 95.3%. The approach employs denoising and wavelet decomposition, two-dimensional modeling, and neural networks for classification, thereby surpassing current methodologies. Sadiq et al. (2019) introduced the multivariate empirical wavelet transform (MEWT) in conjunction with a least-square SVM for classification. Sadiq et al. (2022b) used ReliefF feature selection and multivariate variational mode decomposition strategies for categorization.

Acharya et al. (2012) presented a method for automatic categorization of epileptic EEG activity within a wavelet model. The classification of EEG segments achieved clinically acceptable accuracy while reducing costs through the application of principal component analysis. The Gaussian mixture model classifier achieved 98% accuracy. Akbari et al. (2023) introduced a discrete wavelet transform (DWT) + BPSO for the classification of seizure and seizure-free EEG signals, achieving an accuracy of 99.3%. Gürüler et al. (2014) put forth a categorization approach for obstructive sleep apnea (OSA). Sadiq et al. (2022a) presented a pretrained CNN prototype for the classification of mental imagery and motor EEG signals as well in brain–computer interfaces, demonstrating improved accuracy with ShuffleNet. Nature-inspired feature selection serves as an optimization task aimed at reducing feature space while enhancing accuracy percentages or minimizing prediction errors (Hichem et al., 2022).

Numerous researchers regard feature selection (FSN) as an optimization process due to the extensive search space and intricate interactions within features. FSN approaches are typically categorized into two groups based on their evaluation methods: wrappers and filters.

Metaheuristic algorithms are used to select optimal feature subsets in the literature. The ant-lion optimizer (ALO) (Mirjalili, 2015a) emulates the predatory response of ants and serves as a representative of wrapper-based feature-selection techniques. Grey wolf optimizer (GWO), as detailed by Emily et al., is an effective approach that addresses FSN problems. The study demonstrated effective performance on seven real problems, employing a wrapper-based method known as moth-flame optimization to address feature-selection issues (Mirjalili, 2015b).

Recent research has increasingly adopted attention-based and hybrid deep learning approaches for EEG applications. Wang et al. (2025) reviewed attention mechanisms in brain–computer interfaces, highlighting the advantages of Transformer-based models and attention modules for enhancing spatiotemporal EEG representation. Hyung et al. (2025) demonstrated that cross-attention mechanisms in DeepAttNet significantly improve stress-related EEG classification by capturing interhemispheric neural dependencies. Similarly, Wu et al. (2025) proposed an attention-based multiscale EEGNet, showing that multiscale feature extraction combined with attention yields robust and efficient EEG decoding. These developments underscore the growing importance of attention-enhanced neural architectures and provide motivation for integrating adaptive signal-decomposition techniques with modern learning strategies for improved cognitive-load detection.

In addition, information-theoretic and entropy-based approaches have demonstrated strong effectiveness in assessing stress and neurophysiological load from EEG and ECG signals. Prior studies employed entropy features with genetic-algorithm-based feature selection for emotional-stress detection and Bayesian-optimized KNN using sample entropy for cognitive-stress recognition during mental arithmetic (Sharma et al., 2021a). Approximate entropy (Pincus, 1991) and fuzzy entropy (Mengarelli et al., 2023) have also proven valuable for capturing non-linear neural complexity under fluctuating cognitive demands. Findings from cognitive-stress experiments indicate that mental arithmetic increases frontal brain connectivity and brain–heart coupling, while sustained attention reduces cardiorespiratory interactions, highlighting the integrated and state-dependent nature of neuro-cardiorespiratory coordination under mental stress and cognitive load (Pernice et al., 2021). Furthermore, information-theoretic and LASSO-regularized VAR models have been used to reliably estimate brain–cardiorespiratory interactions under mental stress, even with limited data, demonstrating the value of advanced information-dynamics frameworks for characterizing complex physiological connectivity during cognitive tasks (Antonacci et al., 2020). However, previous studies typically treat workload as a global process and lack spatially resolved analysis. In contrast, the proposed approach combines empirical Fourier decomposition with lobe-wise entropy features and optimized ensemble learning to uncover regional workload markers and enhance neurophysiological interpretability.

The literature review indicates that researchers commonly employ conventional decomposition techniques combined with feature-selection methods. However, these approaches often result in suboptimal accuracy for diverse EEG applications. Among data-adaptive decomposition techniques, empirical mode decomposition (EMD) provides empirical mode extraction but frequently suffers from mode mixing and lacks well-defined frequency boundaries (Wu and Huang, 2009). Empirical wavelet transform (EWT) introduces adaptive spectral segmentation yet remains sensitive to noise and depends on accurate boundary detection (Gilles, 2013). Variational mode decomposition (VMD) offers improved band-limited mode separation but requires pre-selection of the number of modes and penalty parameters, limiting generalizability across subjects and task conditions (Dragomiretskiy and Zosso, 2013). In contrast, EMFD incorporates adaptive spectral thresholding and a zero-phase filter bank, enabling automatic mode selection, eliminating phase distortion, and minimizing mode overlap—resulting in cleaner intrinsic components and enhancing entropy-based characterization of cognitive-load EEG activity. Therefore, this research employs EMFD and optimized machine learning (OML) for signal decomposition, feature extraction, and classification. Furthermore, a lobe-wise analysis is conducted for cognitive-load detection, enabling researchers and clinicians to assess the contributions of different brain regions to cognitive processing and examine spatial variability in workload-related neural responses. The highlights of the proposed approach are:

- To our knowledge, this is the first attempt that uses an EMFD-EEG application for cognitive-load assessment.
- Lobe-wise analysis facilitates the identification of regions exhibiting varying levels of activity under different cognitive loads, resulting in more significant interpretations.
- Lobe-specific classification using EMFD features is novel, enabling both performance gains and neurophysiological interpretability.

## 2 Datasets

**FIGURE 1:**
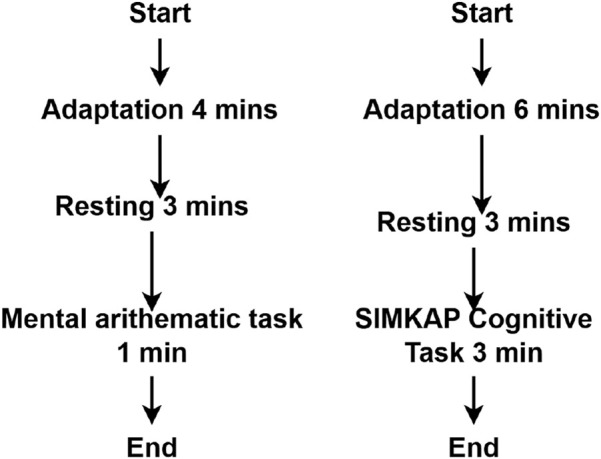
Experimental flows of the MAT and STEW datasets.

This study utilized the publicly accessible MAT and STEW datasets for both lobe-wise and overall cognitive-load identification. Figure 1 illustrates the experimental protocols used for EEG data collection in both datasets. Further details are provided in the following subsections.

### 2.1 MAT

The dataset was recorded from 36 healthy volunteers aged 18–26 years. Arithmetic tasks include serial subtraction and adding two numbers. The EEG signals were recorded at a sampling rate of 500 Hz from individuals using 19 channels (FP1, FP2, Fz, F3, F4, F7, F8, T3, T4, T5, T6, P3, P4, Pz, O1, O2, C3, C4, and Cz) following the 10–20 system electrode placement protocol, both during rest and while performing mental arithmetic tasks. The duration of the recording for rest was 3 min, while for the arithmetic tasks the duration was 1 min (Zyma et al., 2019).

### 2.2 STEW

This dataset was acquired from 48 male college students who performed a single-session simultaneous capacity test (SIMKAP). The participants were instructed to take a rest period while keeping their eyes open for a period of 3 min. Subsequently, a 3-minute-long EEG signal was recorded under workload conditions. The workload includes the SIMKAP test module of the Vienna Test System [34]. The EEG signals were recorded using 14 electrodes (AF3, F7, F3, FC5, T7, P7, O1, O2, P8, T8, FC6, F4, F8, and AF4) and sampled at a rate of 128 Hz (Lim et al., 2018).

## 3 Methods

**FIGURE 2:**
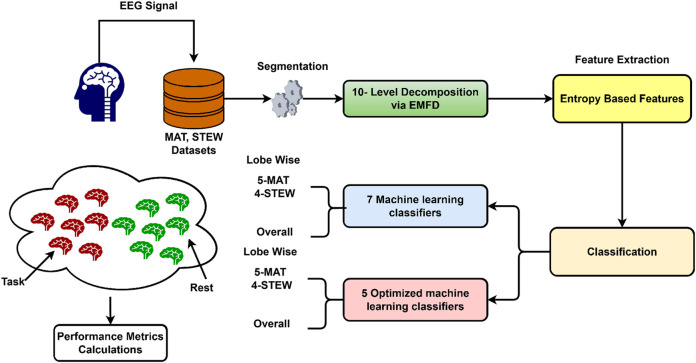
Workflow for cognitive-load detection using EMFD and OML techniques.

The EEG signals were decomposed into intrinsic mode functions (IMFs) using EMFD. Entropy-based features were subsequently derived via these IMFs. The IMFs were classified using several ML and OML classifiers. Figure 2 shows the method employed in this study. The method is explained in detail in the following subsections.

### 3.1 Preprocessing

**FIGURE 3:**
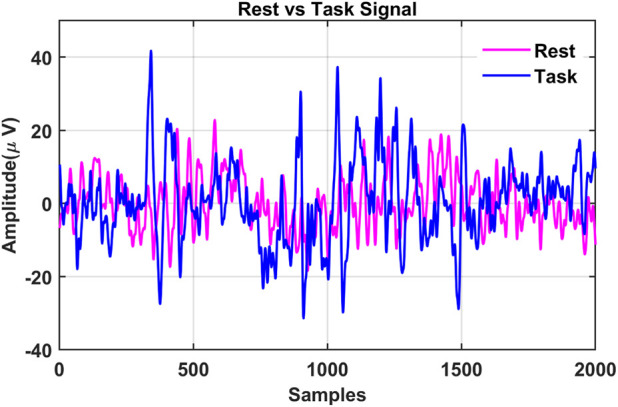
Samples of the 4-s segmented task and rest signals.

During the Preprocessing phase, multiple filters are employed to reduce artifacts and achieve signals devoid of interference. In the MAT dataset, high- and low-pass filters with cutoff frequencies of 0.5 Hz and 45 Hz were utilized to minimize artifacts (Zyma et al., 2019). In contrast, the STEW dataset employed several techniques to expunge artifacts, including a high-pass filter of 1 Hz, eliminating line artifacts, and re-referencing through data averaging (Lim et al., 2018). Samples of the 4-s sliced task and rest signals are illustrated in Figure 3.

### 3.2 EMFD

The EMFD, like the EWT and flow-based decomposition method (FDM), comprises two essential components: an improved segmentation method (IDST) and development of a zero-phase filter bank (ZPFB). EMFD defines the Fourier spectrum (FRSM) of the signal to be sliced within a frequency range of [− π , π ]. Below, we describe IDST and the ZPFB for the spectrum in the frequency of [0, π ].

#### 3.2.1 IDST

An enhanced segmentation approach is formulated, using the lowest minima method as outlined by Gilles et al. (2014). The enhanced segmentation method divides the interval [0, π ] into N contiguous frequency splits. During sorting, the magnitudes of the FRSM at ω = 0 and ω = π , along with their local maxima, are discovered and retrieved. The frequencies associated with the first N largest values in the sorted series are represented as [ $\Omega_{1}$ , $\Omega_{2}$ , $\Omega_{N}$ ]. Furthermore, $\Omega_{0}$ is defined as 0, and $\Omega_{N+1}$ is defined as π . The boundaries of each segment are defined by Equation 1.

$$\omega_{n}=\left\{\begin{array}{cc} \underset{\arg\min\;}{\omega }{\overset{˘}{X}}_{n}\left(\omega \right)\; & \text{ if }0⩽n⩽N\text{ and }\Omega_{n}\neq \Omega_{n+1},\\ \overset{\omega }{\Omega_{n}}\; & \text{ if }0⩽n⩽N\text{ and }\Omega_{n}=\Omega_{n+1}. \end{array}\right.$$ (1)

Here, ${\overset{˘}{X}}_{n}\left(\omega \right)$ is the FRSM magnitudes within $\Omega_{n},\Omega_{n+1}$

#### 3.2.2 ZPFB

Both the EWT and FDM involve crafting a filter bank. The wavelet filter bank is constructed using an empirical scaling procedure along with wavelet functions. A ZPFB is constructed in the EMFD using frequency segments derived from the enhanced segmentation technique. In every frequency portion, a zero-phase filter is a band-pass filter with $\omega_{n-1}$ and $\omega_{n}$ as its cutoff frequencies, characterized by the absence of transition phases. Thus, the ZPFB preserves the primary FRSM component segment while excluding all other FRSM components.

The Fourier transform (FT) of signal x(t) is given by Equation 2.

$$\hat{x}\left(\omega \right)=\int_{-\infty }^{\infty }x\left(t\right)e^{-j\omega t}\;dt.$$ (2)

The ZPFB can be developed via ${\hat{\eta }}_{n}\left(\omega \right)$ is defined by Equation 3.

$${\hat{\eta }}_{n}\left(\omega \right)=\left\{\begin{array}{cc} 1,\; & \text{ if }\omega_{n-1}⩽|\omega |⩽\omega_{n},\\ 0,\; & \text{ otherwise }. \end{array}\right.$$ (3)

The cleaned signal ${\hat{\eta }}_{n}\left(\omega \right)$ is calculated by Equation 4.

$${\hat{x}}_{n}\left(\omega \right)={\hat{\eta }}_{n}\left(\omega \right)\hat{x}\left(\omega \right)=\left\{\begin{array}{c} \hat{x}\left(\omega \right),\text{ if }\omega_{n-1}⩽|\omega |⩽\omega_{n},\;\\ 0,\;\text{ otherwise }.\; \end{array}\right.$$ (4)

The inverse Fourier transform can be utilized to obtain decomposed components in the time domain using the Equation 5.

$$\begin{array}{ll} & x_{n}\left(t\right)=X^{-1}\left[{\hat{x}}_{n}\left(\omega \right)\right]=\int_{-\infty }^{\infty }{\hat{x}}_{n}\left(\omega \right)e^{\text{j}\omega t}\;d\omega =\int_{-\omega_{n}}^{-\omega_{n-1}}\hat{f}\left(\omega \right)e^{\text{j}\omega t}\;d\omega \\ & \;+\int_{\omega_{n-1}}^{\omega_{n}}\hat{f}\left(\omega \right)e^{\text{j}\omega t}\;d\omega . \end{array}$$ (5)

The reconstructed signal is determined by summing all decomposed components using the Equation 6.

$$\tilde{x}\left(t\right)=\overset{N}{\underset{n=1}{\sum }}x_{n}\left(t\right).$$ (6)

**FIGURE 4:**
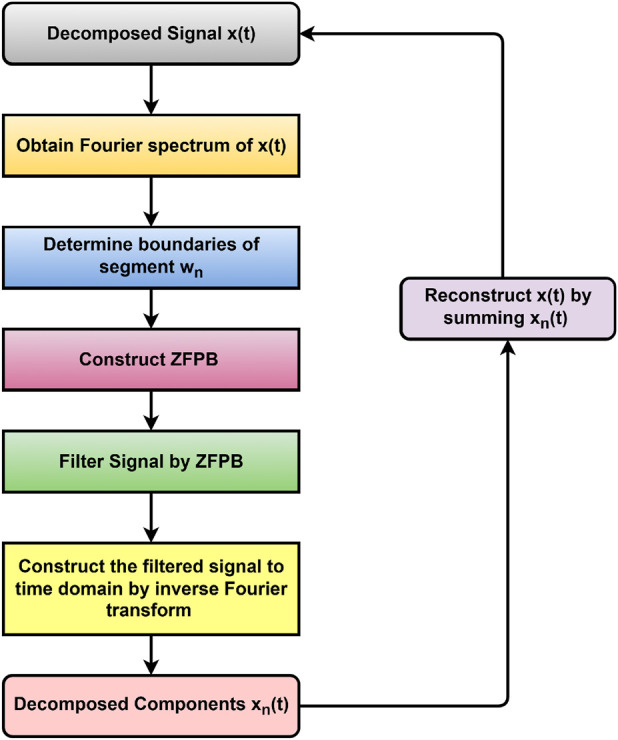
Steps in EMFD

**FIGURE 5:**
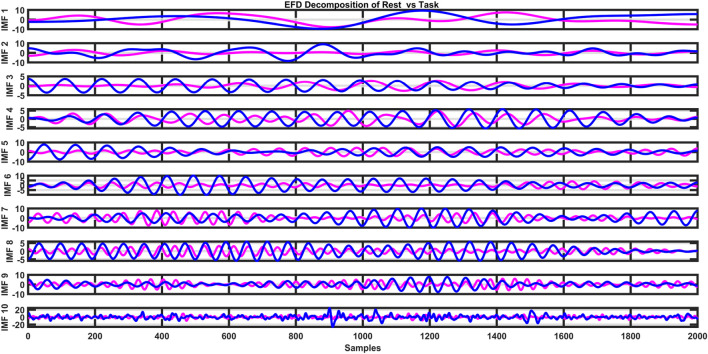
Extraction of modes.

The flow of EMFD is depicted in Figure 4. The IMFs of the decomposed signal are depicted in Figure 5.

#### 3.2.3 Feature extraction

To enable precise classification, feature extraction is necessary to extract significant data from raw EEG signals. In this study, we retrieved entropy-based attributes, including fuzzy entropy ( $F_{e}$ ), approximate entropy ( $A_{e}$ ), differential entropy ( $D_{e}$ ), Renyi’s entropy ( $R_{e}$ ), and fractal dimension entropy ( $FD_{e}$ ), which are delineated in the subsequent subsections.

##### 3.2.3.1 $F_{e}$

Chen et al. proposed the idea of $F_{e}$ . A more effective method corrects the problems with the sample and estimated entropies. When we compare two vectors, we use the Gaussian function instead of the Heaviside function. The Heaviside function’s jumping property distinguishes Shannon entropy from approximation entropy. The subsequent evolution of the Gaussian function allows $F_{e}$ to sidestep the sample’s limitations and approximation entropy. We use Equation 7 to find $F_{e}$ .

$$F_{e}\left(a,b,n,M\right)=\lim_{M\to \infty }\ln\left[\phi^{a}\left(b,n\right)\right]-\ln\left[\phi^{b+1}\left(b,n\right)\right].$$ (7)

##### 3.2.3.2 $A_{e}$

$A_{e}$ refers to unpredictable characteristics of a finite-length signal. It is derived from Kolmogorov entropy. Increasing the phase space embedding factor (M) from M to M+1 requires integrating the signal within the phase area, and the calculation of the degree of variation in the collection of phase space sequences at a specified value (y). Ultimately, $A_{e}$ is represented by Equation 8 (Pincus, 1991):

$$A_{e}\left(N,x,M\right)=\phi \left(x\right)-\phi^{N+1}\left(x\right).$$ (8)

##### 3.2.3.3 $D_{e}$

$D_{e}$ measures the mean unpredictability of a random variable under continuous probability distributions, commonly referred to as continuous entropy and is given by the Equation 9.

F(Y)=−∫h(y)log[h(y)]dy, (9)

where h(y) is the probability density of a random variable y.

For a continuous random variable Y that follows a Gaussian probability density function $h\left(y\right)=N\left(\mu_{Y},\sigma^{2}\right)$ , the differential entropy can be expressed as by Equation 10.

$$H\left(Y\right)=\frac{1}{2}\log\left(2\pi e\;\sigma^{2}\right).$$ (10)

Here, σ denotes the standard deviation of the random variable Y , computed over all realizations as $\sigma =\sqrt{Var\left(Y\right)}$ . This closed-form expression is valid only under the assumption of a Gaussian probability density function (Cover, 1999).

##### 3.2.3.4 $R_{e}$

It was calculated for the random variable $y_{n}$ using Equation 11.

$$IP\left(Y\right)=\frac{1}{P^{2}}\overset{P}{\underset{j=1}{\sum }}\overset{P}{\underset{k=1}{\sum }}B_{\sigma }\left(y_{K}-y_{j}\right),$$ (11)

where $y_{j}$ and $y_{k}$ are dataset samples $j^{th}$ and $k^{th}$ in the Gaussian kernel function $B_{\sigma }\left(y_{K}-y_{j}\right)$ denoted by N.

##### 3.2.3.5 $FD_{e}$

$FD_{e}$ serves as a complexity measure that integrates concepts from fractal computation and information theory to assess the irregularity of time series data, exemplified by EEG signals. Fractal dimension quantifies the extent to which a pattern occupies space. An elevated FD signifies increased complexity or “roughness.” Fractal dimension estimation (FDE) entails the calculation of the fractal dimension for signal segments, followed by the computation of Shannon entropy for the distribution of these fractal dimension values. It is calculated by Equation 12:

$$FD_{e}=-\overset{M}{\underset{i=1}{\sum }}x_{i}log_{2}\left(x_{i}\right).$$ (12)

### 3.3 Classification using ML classifiers

To test the efficacy of our proposed method, we have performed the classification using ML and OML.

The classification using ML involves combining multiple base learners to enhance predictive performance and generalization capability. This technique leverages the strengths of individual models while compensating for their weaknesses through aggregation strategies like bagging, boosting, or stacking. In the context of EEG signal analysis, ensemble classifiers are particularly effective at handling the non-linearity and high-dimensionality of EEG features, resulting in improved classification robustness and accuracy.

Classification using OML entails fine-tuning algorithms to attain peak performance for designated tasks. This process involves feature selection, hyperparameter tuning, and strategies to mitigate overfitting. OMLs enhance $A_{c}$ and efficiency of categorizing different states in EEG signal analysis, based on features extracted.

## 4 Results

The suggested method was tested in MATLAB R2022a on a system featuring an Intel Core i7 processor with 16 GB RAM. Classification is done by using ensemble classifiers and optimized ensemble classifiers, lobe-wise for both datasets and the overall datasets. In this work, we have performed the overall and lobe-wise classification, and the effectiveness of the propsed method was evaluated using standard performance metrics, such as accuracy ( $A_{c}$ ), sensitivity ( $S_{e}$ ), specificity ( $S_{p}$ ), precision ( $P_{r}$ ), and F-score ( $F_{s}$ ) (Chaitanya and Sharma, 2024).

### 4.1 Overall classification

The performance metrics obtained for the MAT and STEW datasets using ML are presented in Tables 1, 2, respectively. Table 1 shows that we obtained the best $A_{c}$ of 95.8%, $S_{e}$ of 96.4%, and $S_{p}$ of 95.10% using the ensemble boosted tree classifier for the MAT dataset. Similarly, from Table 2, we observe that for the STEW dataset, the best $A_{c}$ of 95%, $S_{e}$ of 95.4%, and $S_{p}$ of 98.9% are obtained using ensemble boosted tree classifier.

**TABLE 1:** Outcomes of the proposed method for overall classification using the MAT dataset by ML.

Classifier	$A_{c}$ (%)	$S_{e}$ (%)	$S_{p}$ (%)	$P_{r}$ (%)	$F_{s}$ (%)
Fine tree	85.10	84.26	85.93	85.69	84.96
Fine tree (FTE)	85.10	84.26	85.93	85.69	84.96
Medium tree (MT)	84.30	83.70	84.81	84.64	84.11
Coarse tree (CT)	73.20	67.96	78.52	75.98	71.74
Linear discriminant (LD)	51.70	6.48	96.85	67.31	11.82
Quadratic discriminant (QD)	54.30	8.89	99.63	96.00	16.27
Binary GLM logistic regression (BGLR)	56.00	53.33	58.70	56.36	54.75
Efficient logistic regression (ELR)	50.80	42.90	58.70	50.99	46.62
Efficient linear SVM (ELS)	52.00	53.15	50.93	51.99	52.56
Gaussian naïve Bayes (GNB)	50.60	2.96	98.33	64.00	5.60
Kernel naïve Bayes (KNB)	54.30	6.48	78.52	84.64	16.27
Linear SVM (LS)	84.90	84.60	85.19	85.10	84.90
Quadratic SVM (QS)	91.70	90.19	93.10	92.90	91.40
Cubic SVM (CS)	92.90	91.10	94.60	94.40	92.70
Fine Gaussian SVM (FGS)	76.20	87.40	65.00	71.40	78.40
Medium Gaussian SVM (MGS)	89.20	87.50	90.70	90.44	88.99
Coarse Gaussian SVM (CGS)	75.60	78.30	72.90	74.30	76.28
Fine KNN (FK)	53.50	83.30	98.70	86.50	15.21
Medium KNN (MK)	53.10	6.40	99.60	94.50	12.10
Coarse KNN (COK)	51.50	5.00	97.90	71.05	9.30
Cosine KNN (CSK)	52.90	6.80	98.80	86.04	12.60
Cubic KNN (CUK)	52.30	5.70	98.80	83.70	10.70
Weighted KNN (WK)	53.70	7.90	99.40	93.40	14.60
Ensemble boosted trees (EBT)	**95.80**	**96.40**	**95.10**	**95.20**	**95.80**
Bagged trees (BT)	93.40	92.70	94.00	94.00	93.00
Subspace discriminant (SDT)	54.20	8.33	100.00	100.00	15.00
Subspace KNN (SK)	93.90	93.30	94.40	94.38	93.70
Rusboosted trees (RT)	83.70	82.70	84.60	84.30	83.50

Bolded values indicate statistically significant results.

**TABLE 2:** Outcomes of the proposed method for overall classification using the STEW dataset by ML.

Classifier	$A_{c}$ (%)	$S_{e}$ (%)	$S_{p}$ (%)	$P_{r}$ (%)	$F_{s}$ (%)
FT	82.10	83.26	81.93	82.69	80.96
FTE	81.10	82.26	81.30	85.90	84.60
MT	74.30	73.70	64.81	74.64	74.11
CT	70.20	68.96	79.52	74.98	70.74
LD	55.70	46.48	86.85	57.31	71.82
QD	56.30	58.89	96.63	95.00	86.27
BGLR	56.50	50.33	57.70	57.36	53.75
ELR	51.80	52.90	59.70	51.90	49.62
ELS	52.00	53.15	50.93	51.99	52.56
GNB	51.60	52.96	78.33	66.00	55.60
KNB	54.80	56.48	70.52	74.64	76.27
LS	86.90	85.60	81.19	84.10	81.90
QS	90.70	90.19	91.10	90.90	90.40
CS	92.70	91.40	94.90	94.00	92.00
FGS	74.20	83.40	66.00	70.40	75.40
MGS	85.20	85.50	92.70	91.44	85.99
CGS	75.60	78.30	71.90	75.30	74.28
FK	51.50	82.30	97.00	86.00	55.21
MK	54.10	56.40	95.60	94.50	92.10
COK	51.50	5.00	97.90	71.05	9.30
CSK	52.90	6.80	98.80	86.04	12.60
CUK	53.30	53.70	58.80	53.70	10.70
WK	53.70	57.90	59.40	90.40	44.60
EBT	**95.00**	**95.40**	**98.90**	**96.20**	**95.80**
BT	94.40	91.70	93.00	94.00	94.00
SDT	54.20	8.33	91.10	93.20	90.10
SK	93.90	93.30	94.40	94.38	93.70
RT	83.50	82.31	84.00	83.30	84.50

Bolded values indicate statistically significant results.

**FIGURE 6:**
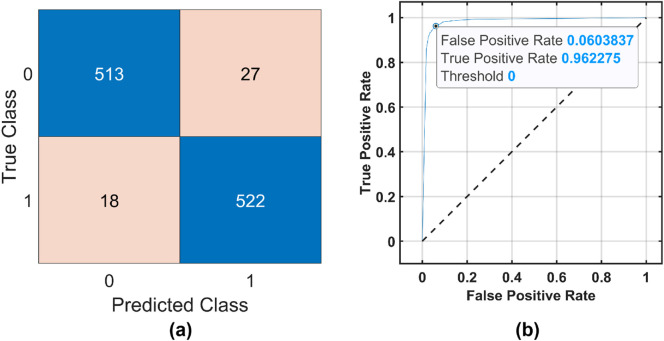
Ensemble boosted tree performance for the MAT dataset. **(a)** Confusion Matrix and **(b)** ROC Curve .

**FIGURE 7:**
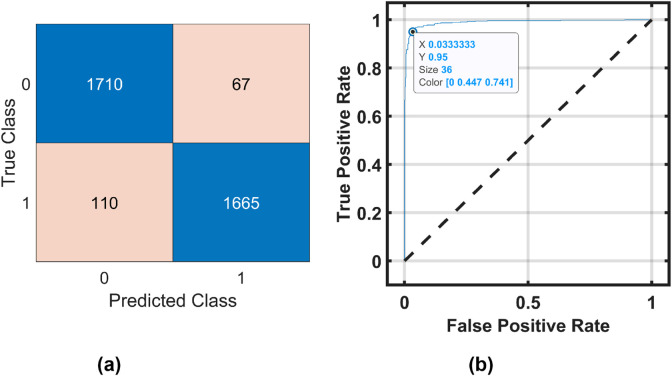
Ensemble boosted tree performance for the STEW dataset. **(a)** Confusion Matrix and **(b)** ROC Curve .

Figures 6, 7 show the confusion matrix and receiver operating characteristic (ROC) curve obtained by the ensemble boosted tree using the MAT and STEW datasets, respectively.

**FIGURE 8:**
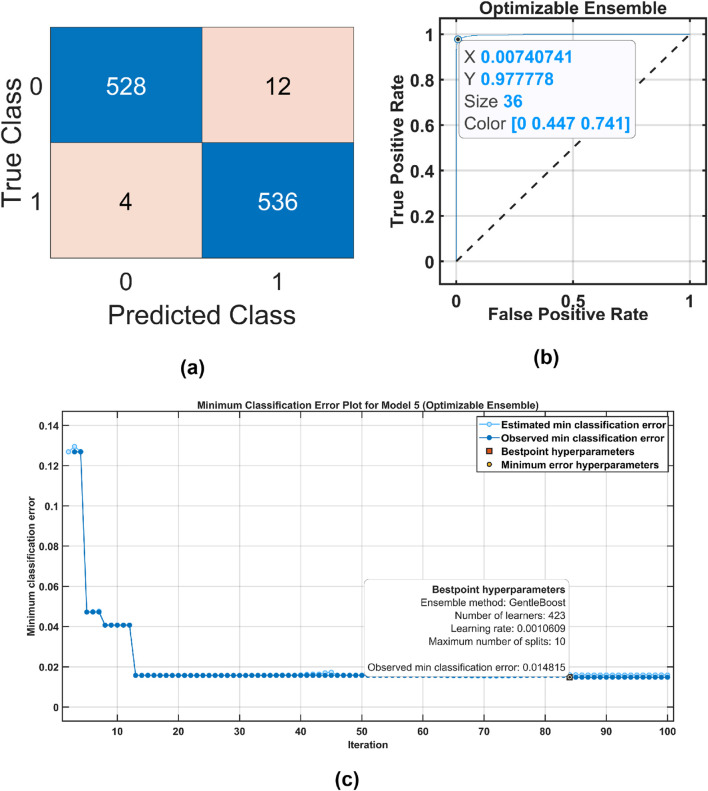
Optimized ensemble classifier performance for the MAT dataset. **(a)** Confusion Matrix, **(b)** ROC Curve, and **(c)** Classification error plot .

**FIGURE 9:**
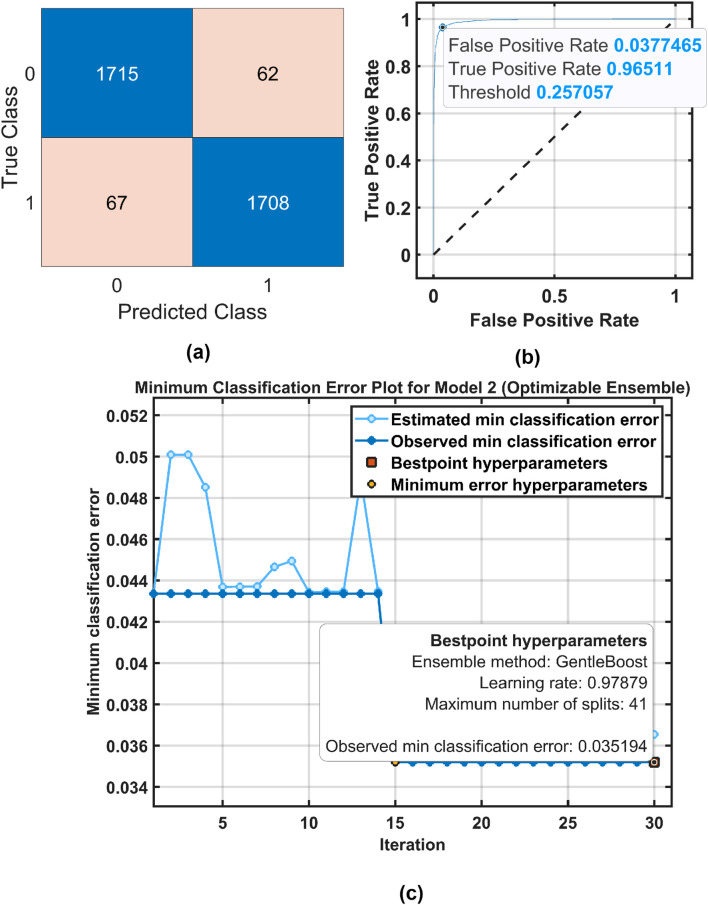
Optimized ensemble classifier performance for the MAT dataset. **(a)** Confusion Matrix, **(b)** ROC Curve, and **(c)** Classification error plot .

The performance metrics obtained for the MAT and STEW datasets using OMLs are presented in Tables 3, 4. Table 3 shows that we obtained the best $A_{c}$ of 97.8%, $S_{e}$ of 98.5%, and $S_{p}$ of 97.4% using OML. Similarly, Table 4 shows that we obtained the best $A_{c}$ of 96.3%, $S_{e}$ of 96%, and $S_{p}$ of 96.57% using OML. Figures 8, 9 show the confusion matrices, ROC, and classification error plots obtained by the OMLs using the MAT and STEW datasets.

**TABLE 3:** Outcomes of the proposed method for overall classification using the MAT dataset by OMLs.

Classifier	$A_{c}$ (%)	$S_{e}$ (%)	$S_{p}$ (%)	$P_{r}$ (%)	$F_{s}$ (%)
Optimizable ensemble (OE)	**97.80**	**98.15**	**97.40**	**97.43**	**97.79**
Optimizable KNN (OKN)	93.90	93.10	94.63	94.55	93.85
Optimizable naïve Bayes (ONB)	69.60	67.04	72.22	70.70	68.82
Optimizable SVM (OSM)	88.20	87.04	89.44	89.13	88.01
Optimizable discriminant (OD)	54.30	9.07	99.44	94.23	16.55
Optimizable tree (OT)	86.00	85.00	87.04	86.77	85.83

Bolded values indicate statistically significant results.

**TABLE 4:** Outcomes of the proposed method for overall classification using the STEW dataset by OMLs.

Classifier	$A_{c}$ (%)	$S_{e}$ (%)	$S_{p}$ (%)	$P_{r}$ (%)	$F_{s}$ (%)
OE	**96.30**	**96.00**	**96.57**	**96.54**	**96.30**
OKN	51.90	23.32	80.42	54.33	32.67
ONB	71.80	60.90	82.61	77.77	68.30
OSM	90.70	88.96	92.40	92.12	90.50
OD	55.30	90.70	95.44	94.30	16.65
OT	84.00	84.00	83.68	83.79	84.11

Bolded values indicate statistically significant results.

**FIGURE 10:**
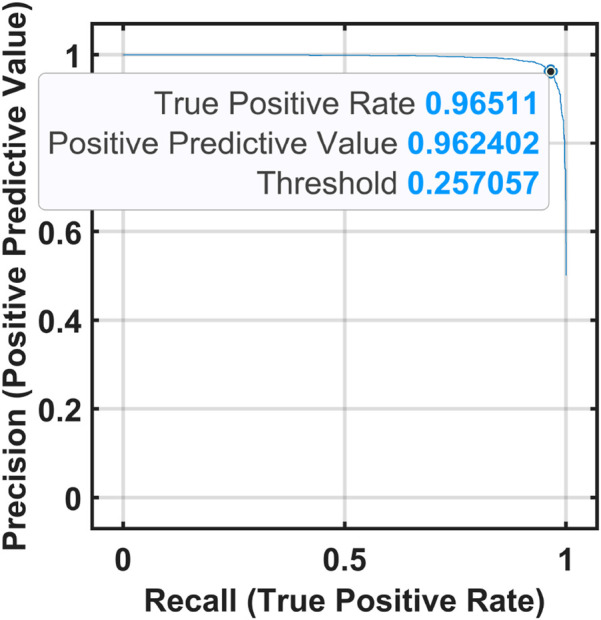
Graph showing precision vs. recall for the STEW dataset.

**FIGURE 11:**
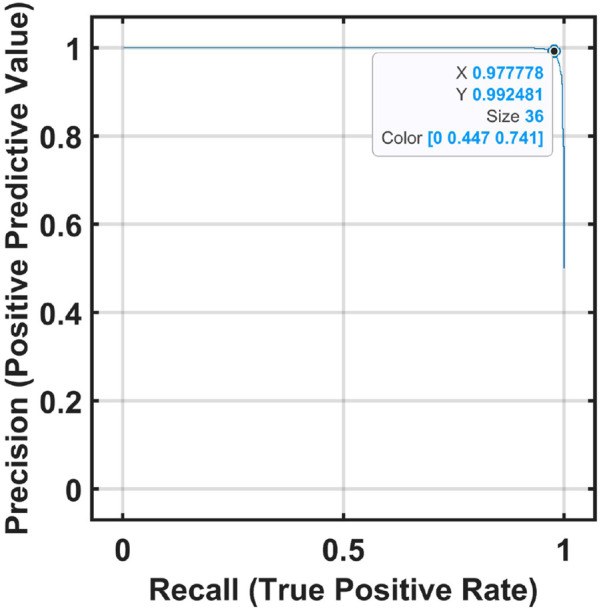
Graph showing precision vs. recall for the MAT dataset.

Graphs showing the precision and recall obtained for the STEW and MAT datasets are presented in Figures 10, 11.

#### 4.2 Lobe-wise classification

EEG analysis frequently investigates brain activity concerning the primary cortical lobes, each of which is linked to specific functional specializations. The frontal lobe, situated at the anterior region of the brain, regulates advanced cognitive functions, including decision-making, attention management, working memory, problem-solving, and voluntary motor activities. The parietal lobe, located in the upper posterior region, integrates multisensory inputs and is essential for spatial orientation and proprioception. The temporal lobe, located laterally near the ears, plays a crucial role in auditory perception, language comprehension, and memory processing. The occipital lobe, located at the posterior end of the brain, is primarily responsible for processing visual information. The international 10–20 electrode placement system designates specific electrode sites that correspond to distinct lobes, facilitating the targeted examination of neural activity patterns specific to each lobe.

We performed lobe-wise classification for the data in the MAT and STEW datasets using ML methods and have presented the best outcomes in Table 5. Table 5 shows that we obtained the best $A_{c}$ of 94.2%, $S_{e}$ of 95.06%, and $S_{p}$ of 93.42% for the parietal lobe of MAT dataset using ensemble boosted tree classifiers and $A_{c}$ of 96.2%, $S_{e}$ of 94.99%, and $S_{p}$ of 97.41% for the occipital lobe of the STEW dataset using a subspace KNN classifier.

**TABLE 5:** Outcomes of the proposed method for lobe-wise classification using OMLs.

Classifier	Lobe	$A_{c}$ (%)	$S_{e}$ (%)	$S_{p}$ (%)	$P_{r}$ (%)	$F_{s}$ (%)	Dataset
EBT	Central	89.40	87.80	90.90	90.60	89.23	MAT
EBT	Frontal	**94.20**	**95.06**	**93.42**	**93.52**	**94.28**	
EBT	Occipital	88.00	87.04	88.89	88.68	87.83	
EBT	Parietal	92.80	92.18	93.42	93.33	92.73	
CS	Temporal	80.20	80.60	79.80	80.00	80.30	
WK	Frontal	**96.20**	**94.99**	**97.41**	**97.35**	**96.13**	STEW
SK	Occipital	94.14	93.13	95.16	95.05	94.00	
SK	Parietal	95.80	95.77	95.78	95.77	95.77	
CS	Temporal	88.93	88.92	88.95	89.00	89.01	

Bolded values indicate statistically significant results.

The best outcomes of performing lobe-wise classification using all the OMLs are presented in Tables 6, 7. Table 6 shows that we obtained the best $A_{c}$ of 97.8%, $S_{e}$ of 98.3%, and $S_{p}$ of 97.2% for the frontal lobe of the MAT dataset using optimizable ensemble classifiers. Table 7 shows that we obtained the best $A_{c}$ of 96.08%, $S_{e}$ of 95.9%, and $S_{p}$ of 96.27% for the frontal lobe of the MAT dataset using an optimizable ensemble classifier. The ROC curves in Figures 6, 7 show the proposed EMFD-based entropy framework with optimized ensemble learning on the MAT and STEW datasets. The curves demonstrate high discriminative performance for cognitive-load classification across subjects, with area under the curve (AUC) values approaching 1, indicating excellent sensitivity and specificity. The steep rise of the ROC curves toward the top-left corner reflects strong separability between low and high load conditions, validating the effectiveness of the lobe-wise feature extraction and classification strategy.

**TABLE 6:** Outcomes of the proposed technique for lobe-wise classification using the MAT dataset by OMLs.

Classifier	Lobe	$A_{c}$ (%)	$S_{e}$ (%)	$S_{p}$ (%)	$P_{r}$ (%)	$F_{s}$ (%)
OE	Occipital	96.30	96.85	95.74	95.79	96.27
OKN		81.20	82.90	79.44	80.10	81.54
ONB		70.93	71.67	70.19	70.69	71.17
OSM		84.80	82.40	87.20	86.60	84.40
OD		52.20	63.00	98.10	77.20	11.60
OT		81.30	82.20	80.30	80.70	81.40
OE	Central	96.30	97.04	95.56	95.52	96.27
OKN		84.80	79.63	90.00	88.80	84.18
ONB		71.20	72.04	70.37	70.86	71.44
OSM		86.90	87.40	86.40	86.60	87.01
OD		52.30	7.96	96.67	70.49	14.29
OT		82.50	82.90	82.04	82.20	82.58
OE	Temporal	86.40	84.81	87.96	87.57	86.11
OKN		73.40	70.70	76.11	74.76	72.68
ONB		62.50	59.26	65.74	63.37	61.21
OSM		80.60	80.19	81.11	80.93	80.55
OD		52.10	6.11	98.15	76.74	11.34
OT		74.20	74.07	74.26	74.12	74.09
OE	Frontal	**97.80**	**98.30**	**97.20**	**97.20**	**97.70**
OKN		90.80	91.30	90.30	90.40	90.80
ONB		70.50	67.50	73.30	71.70	69.50
OSM		93.30	90.30	96.30	96.06	93.11
OD		53.40	8.50	98.30	83.60	15.40
OT		84.20	85.10	83.30	83.60	84.30
OE	Parietal	97.78	97.41	97.96	97.95	97.68
OKN		93.30	94.20	92.40	92.50	93.40
ONB		72.50	72.00	72.90	72.70	72.30
OSM		88.20	89.40	87.04	87.35	88.38
OD		56.02	14.40	97.50	85.70	24.50
OT		82.90	82.70	82.90	82.90	82.80

Bolded values indicate statistically significant results.

**TABLE 7:** Outcomes of the proposed technique for lobe-wise classification using the STEW dataset by OMLs.

Classifier	Lobe	$A_{c}$ (%)	$S_{e}$ (%)	$S_{p}$ (%)	$P_{r}$ (%)	$F_{s}$ (%)
Occipital	OE	95.44	95.10	95.78	95.75	95.42
	OKN	95.90	95.04	96.74	96.68	95.72
	ONB	72.21	69.13	75.29	73.65	71.32
	OSM	94.14	93.01	95.27	95.16	94.08
	OD	51.20	63.00	91.10	74.20	18.60
	OT	83.05	83.94	82.16	82.46	83.18
Temporal	OE	91.42	91.35	91.49	91.48	91.41
	OKN	84.20	75.52	92.85	91.35	82.66
	ONB	74.00	72.76	75.24	74.57	73.65
	OSM	85.94	86.56	85.22	85.40	85.98
	OD	53.10	64.11	92.15	75.74	17.34
	OT	79.33	80.73	77.94	78.52	79.66
Frontal	OE	**96.08**	**95.90**	**96.27**	**96.28**	**96.11**
	OKN	93.20	89.80	96.57	96.31	92.92
	ONB	71.00	58.25	83.74	78.16	66.76
	OSM	92.40	91.09	93.69	93.52	92.40
	OD	51.40	18.50	94.30	82.60	17.40
	OT	80.24	80.34	80.14	80.16	80.24
Parietal	OE	94.89	94.76	95.05	94.97	94.87
	OKN	94.20	94.19	94.26	94.25	94.22
	ONB	73.13	73.00	73.66	73.66	72.99
	OSM	92.50	91.99	92.89	92.84	92.41
	OD	55.02	54.40	90.50	86.70	25.50
	OT	81.40	81.69	81.01	81.15	81.42

Bolded values indicate statistically significant results.

**FIGURE 12:**
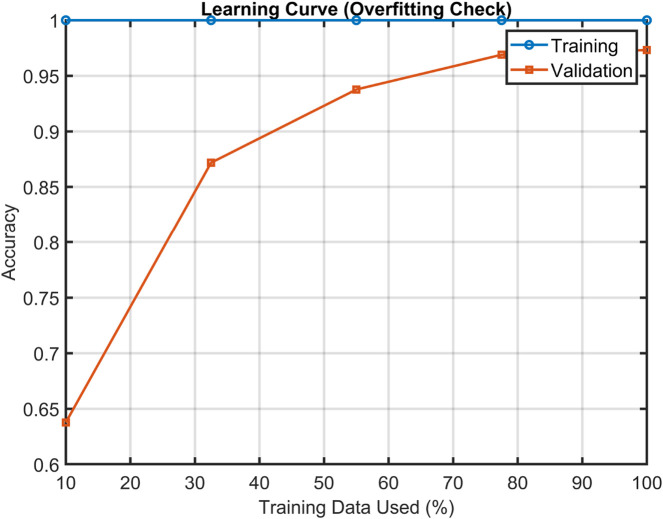
Learning curve for EEG-based cognitive load classification.

To evaluate the robustness and generalization capability of the proposed model, a 10-fold cross-validation strategy (Antonacci et al., 2024) was adopted using a gentle boost ensemble classifier. The model achieved a mean classification accuracy of 97.8%, with a standard deviation of 0.013 and a 95% confidence interval of [96.8%, 98.7%] across folds, indicating high performance consistency. In addition, a permutation test with 100 iterations was performed to assess the likelihood of achieving similar accuracy by chance. The resulting p-value < 0.001 confirms that the observed performance is statistically significant and not a result of random label associations. Furthermore, a learning-curve analysis was conducted to examine the effect of training-data proportion on model performance. As shown in Figure 12, the validation accuracy improves steadily with increasing training data, while training accuracy remains consistently high, suggesting genuine generalization capability and ruling out overfitting risk. These collective findings demonstrate the reliability and stability of the proposed EEG-based cognitive-load classification framework.

## 5 Discussion

Table 8 compares the method proposed in this study with prior machine learning models. The discrete wavelet transform (DWT) is widely utilized by researchers in statistical signal processing, especially in areas such as EEG analysis. Scientists employ SWT to address DWT’s deficiency. This approach was deemed superfluous as the sample count at every stage of the SWT output matches the sample count of the input. An application of empirical mode decomposition is the Hilbert–Huang transform (HHT). This method retrieves time–frequency data from a signal characterized by chaotic behavior and temporal variability (Hasan and Kim, 2019; Vanitha and Krishnan, 2016; Gupta et al., 2020). The primary function of this classifier is to decompose an EEG signal into distinct modes. This method exhibits increased complexity relative to alternative approaches owing to the occurrence of mode mixing. The technique accommodates various resolutions and employs data for signal detection. This system exhibits high sensitivity to noise, and the occurrence of mode mixing may exacerbate the situation. The attributes derived from the EEG data significantly impact classification accuracy. This research utilized the EMFD method for signal decomposition. It breaks down signals into modes, with each mode corresponding to a distinct frequency component. This multiscale parsing captures low- and high-frequency EEG instances associated with cognitive stress, facilitating the detection of subtle variations in brain activity. Six machine learning classifiers were employed, utilizing K-fold cross-validation with a 10-fold configuration. Ten-fold cross-validation offers a sturdy, productive, trustworthy method for assessing model performance. This approach effectively balances the necessity for precise outcomes estimation with computational practicality, rendering it a favored option among numerous machine learning aspirants. They enhance accuracy, mitigate overfitting, and require hyperparameter tuning.

**TABLE 8:** Comparison of the proposed approach with existing approaches.

Reference	Year	Dataset	Technique	$A_{c}$ (%)	$S_{e}$ (%)	$S_{p}$ (%)
Sharma et al. (2022)	2012	MAT	SWT + WOA + SVM	97.25	97.20	98.10
Al-Shargie et al. (2015)	2016	MAT	WT + SVM	93.36	92.59	94.71
Vanitha and Krishnan (2016)	2017	MAT	HHT + SVM	89.07	90.12	89.00
Cheema and Singh (2019)	2019	STAI	EMD + SVM	93.14	92.00	94.44
Hasan and Kim (2019)	2020	DEAP	DWT + KNN	96.60	97.00	96.80
Gupta et al. (2020)	2020	NEMAR	DCT + BPSO + SVM	96.36	96.85	90.80
Sharma et al. (2021b)	2021	MAT	SWT + KNN	96.00	96.72	95.62
Sharma et al. (2021a)	2021	MAT	SWT + optimized KNN	94.00	92.10	94.83
Baygin et al. (2023)	2023	MAT	Pooling function + SVM	96.42	96.11	97.52
Jain et al. (2024)	2024	MAT	VMD + LGBM	97.20	97.40	96.90
Havugimana et al. (2023)	2024	Bootstrap + GAN	CNN	94.00	93.20	95.10
Yedukondalu et al. (2025c)	2025	MAT	R-LMD + BAO + EL	97.40	97.00	98.00
**Proposed (MAT)**	**2025**	**MAT**	**EMFD + OML**	**97.80**	**98.15**	**97.40**
**Proposed (STEW)**	**2025**	**STEW**	**EMFD + OML**	**96.40**	**96.00**	**96.50**

Bolded values indicate statistically significant results.

**FIGURE 13:**
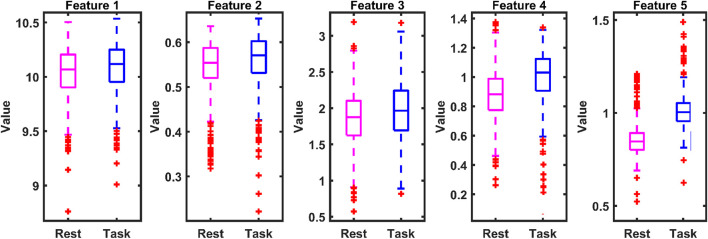
Box plot of the top five features of the frontal lobe in the MAT dataset.

Figure 13 presents boxplots of the top three lobes demonstrating superior classification performance of the proposed approach on the MAT dataset, as reported in Table 1. These plots illustrate the interclass distribution of the selected features, showing that the task-related signals yield higher values than the baseline conditions. Additionally, boxplots of classification accuracy across multiple runs on both the MAT and STEW datasets confirm the stability of the EMFD-based entropy features combined with optimized ensemble learning. The narrow interquartile ranges, minimal variance, and negligible outliers indicate strong model consistency across subjects and trials, demonstrating the robustness of the proposed framework for reliable cognitive-load detection at both lobe-wise and overall levels.

**FIGURE 14:**
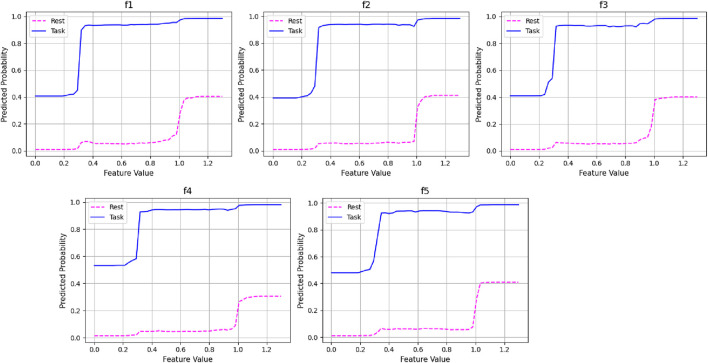
Dependency plot of the top five features of the frontal lobe in the MAT dataset.

Dependency plots obtained from the proposed method are illustrated in Figure 14. From Figure 14, the top features f1 to f5, derived from EEG signals, demonstrate a consistent pattern: lower values of these features are associated with a higher probability of the “Task” class, while higher values—especially beyond a threshold of approximately 0.4—shift the model’s prediction toward the “Rest” class. This threshold-like behavior indicates the ensemble model’s ability to capture non-linear decision boundaries, leveraging clear cutoffs in feature values for robust classification. The distinct and well-separated probability curves for both classes reflect the strong discriminative power of the selected EEG features. Such interpretability is crucial in EEG-based applications, as it highlights how specific brain signal characteristics contribute to class separation and supports model transparency in cognitive state monitoring.

The proposed method’s lobe-wise findings are aligned with established cortical specialization in cognitive neuroscience. The frontal lobe showed the highest discrimination performance in both datasets, which reflects its role in executive control, attention regulation, working memory, and mental arithmetic processing (Miller and Cohen, 2001; Antonenko et al., 2010). Frontal theta enhancement and alpha modulation have long been associated with cognitive effort and attentional control (Klimesch, 2012). Parietal-lobe performance aligns with its involvement in multimodal sensory integration and spatial-attention control within the fronto-parietal network (D’Esposito and Postle, 2015; Corbetta and Shulman, 2002). The strong occipital contribution in visually demanding tasks, as seen in STEW, corresponds to well-known visual-processing and visuospatial workload pathways (Kulasingham et al., 2021). Furthermore, the proposed method results are consistent with studies demonstrating the utility of entropy-based neural features in cognitive and emotional-stress monitoring. Entropy measures capture subtle fluctuations in neural complexity under workload (Shon et al., 2018; Sharma et al., 2021b). ROC curves demonstrated strong discriminatory ability, with AUC values approaching 1.0, confirming that the EMFD-based entropy features and optimized ensemble model effectively distinguish between low- and high-cognitive-load states. Box plot analysis further indicated narrow interquartile ranges and minimal variability across runs, highlighting the stability and reliability of the proposed framework across subjects and trials. In addition, dependency plots illustrated consistent relationships between key entropy features and predicted cognitive-load levels, providing further insight into feature contribution and model decision behavior. By applying these metrics to lobe-wise empirical Fourier modes, we provide a novel fusion of information-theoretic analysis and neurophysiological interpretability, yielding insights into localized cortical dynamics during cognitive load.

## 6 Conclusion

The presented research presents innovative methods for calculating multi-domain attributes to assess CL in two datasets: MAT and STEW. Two instances for examination were utilized: lobe-wise and overall. EEG signals were sliced and decomposed into five modes via EMFD, from which entropy-based features were extracted. Six machine learning prototypes were optimized, trained, and assessed through 10-fold cross-validation. Overall analysis yielded a maximum classification accuracy of 97.8% for the MAT and 96.4% for the STEW datasets. Lobe-wise analysis utilized five lobes for MAT and four lobes for STEW, with the frontal lobe demonstrating the highest accuracy of 97.8% and 96.08%, respectively. The suggested approach (EMFD + OML) exhibited enhanced efficacy over previous methods in the identification of CL via decomposition and feature extraction.

## 7 Future scope

The following limitations of the proposed framework highlight promising directions for future research in cognitive-load detection:

- The current study relies on manually engineered features from EMFD-derived IMFs. Future work will explore automated feature-learning strategies, such as deep learning-based representations, to enhance model robustness, reduce manual intervention, and further improve classification accuracy.
- Additional signal preprocessing and artifact-removal pipelines will be incorporated to strengthen noise resilience and overall model stability.
- Further investigations will evaluate other data-driven signal-decomposition techniques and advanced feature-selection strategies to identify the most informative cognitive-load biomarkers.
- This study utilizes two EEG datasets from 84 participants. Future studies will incorporate larger and more diverse datasets with broader demographic representation and varied cognitive tasks to improve generalizability and strengthen real-world applicability.

## Data Availability

The original contributions presented in the study are included in the article/Supplementary Material, further inquiries can be directed to the corresponding author.
